# COVID‐19 and Pregnancy: A Case Study

**DOI:** 10.1002/gch2.202000074

**Published:** 2021-05-13

**Authors:** Rui Long, Di Wu, Xingguang Lin, Dan Lv, Renjie Wang, Lei Jin, Shujie Liao, Weiliang Liu, Dongrui Deng

**Affiliations:** ^1^ Department of Obstetrics Tongji Hospital Tongji Medical College Huazhong University of Science and Technology Wuhan 430030 China; ^2^ Department of Obstetrics Caidian District People's Hospital Wuhan 430100 China

**Keywords:** birth induction, COVID‐19, IgG, vertical transmission

## Abstract

The 2019 novel coronavirus disease is spreading all over the world. Pregnant women and infants require particular concern, owing to the special immune conditions. A case of a pregnant woman who was exposed to SARS‐CoV‐2 at 34+1 weeks gestation and chose to continue pregnancy is reported. Without obvious symptoms or signs, the woman did not receive any treatment before delivery, and gave birth at 37+5 weeks to a neonate with positive immunoglobulin G for SARS‐CoV‐2 and negative nucleic acid tests. The mother was given anti‐infection, oxytocin, and fluid rehydration treatment after delivery. Both mother and infant recovered well after a three‐month follow‐up. Continued expectation to deliver at term instead of preterm can decrease the potential risk of severe perinatal and infant complications and is beneficial to the development of the neonate. More studies are required to confirm the presence of vertical transmission.

## Graphical abstract

A case study of a pregnant woman who was exposed to SARS‐CoV‐2 at 34+1 weeks gestation and continued expectation until 37+5 weeks with a healthy neonate is reported.

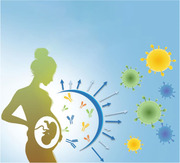

The World Health Organization warned that the 2019 novel coronavirus disease (COVID‐19) outbreak can be characterized as a “pandemic” given that the virus spreads increasingly worldwide. More than 39 million people have been diagnosed across the world till October 16, 2020, with a death toll surpassing 1,099,000.^[^ ^1^ ^]^ Pregnant women and neonates may have a high risk of infecting COVID‐19, owing to their special immune states.^[^ ^2^ ^]^ Limited studies have reported that pregnant women who were infected by other virus delivered immediately due to deteriorating maternal condition, and observed respiratory complications or stillbirth.^[^ ^3^ ^]^ However, for pregnant women with COVID‐19, there is no direct evidence to verify whether inducing birth immediately can decrease the risk of adverse outcome.

In this study, we report a confirmed case of pregnant woman who was exposed to severe acute respiratory syndrome coronavirus 2 (SARS‐CoV‐2) at 34+1 weeks and gave birth at 37+5 weeks to a neonate with positive immunoglobulin G (IgG) for SARS‐CoV‐2.

A 30‐year‐old woman (gravida 2, para 1) at 34+1 weeks’ gestation developed fever on February 2, 2020. The highest point reached 38.5 °C. Before the onset of the symptom, nuchal translucency (NT), maternal serum markers for Down's Syndrome screening, noninvasive prenatal genetic testing (NIPT), oral glucose tolerance test (OGTT), and fetal ultrasound were performed on the patient, and no abnormity was found. She lived with her husband, her son, and parents‐in‐law and had no other close contact expect her family members. On the same day, her mother‐in‐law, who always went to market with nonsurgical mask and eventually diagnosed with COVID‐19, developed the same symptom. After developing fever, the patient started to take Ibuprofen suspension (20 mL PO Q8hrs) by herself and relieved after 8 d.

All family members received nucleic acid tests (NATs) for SARS‐CoV‐2 on throat swab samples on February 8, 2020, and positive result was found only in her mother‐in‐law. However, abnormal chest computed tomography (CT) was noticed in all members except her son. Ground‐glass opacities were found in the patient's right‐side lung.

Then the patient was transferred to isolation ward as a close contact with the confirmed case. The nucleic acid test was repeated twice but both were negative. Without obvious symptoms or signs, she did not receive any treatment in the isolation ward and chose to continue expectation till 37+5 weeks without fully maturation treatment. Fetal ultrasound on February 22, 2020, found no abnormality.

Before delivery, her vital signs were normal (body temperature 36.5 °C, blood pressure 130/80 mmHg, respiratory rate 20 breaths min^−1^, and pulse rate 80 beats min^−1^), and the pertinent laboratory findings are listed in **Table** **1**. The fetal heart rate (FHR) was 135 bpm and fetal heart monitoring was normal. Due to the history of the scarred uterus, the patient underwent a cesarean section at 37+5 weeks’ gestation on February 27, 2020. A 3860 g male infant was delivered without complication. Apgar scores at 1 and 5 min were 8 and 9, respectively. Lung auscultation on neonate revealed no rhonchi over the lung. The mother was given anti‐infection, oxytocin, fluid rehydration treatment after surgery. Laboratory examinations revealed elevated leukocyte count, neutrophils, and C‐reactive protein level (listed in Table 1). She was afebrile without any discomfort. Her pharyngeal swab sampled for COVID‐19 was still negative on two successive examinations and the result of CT was normal on March 2, 2020. The mother was discharged on March 5, 2020, and a high level of IgG and immunoglobulin M (IgM) to SARS‐CoV‐2 was first found from the peripheral blood on March 10, 2020. Therefore, according to the *Chinese Clinical Guidance for COVID‐19 Pneumonia Diagnosis and Treatment* (7th ed.),^[^ ^4^ ^]^ this patient was finally diagnosed as a COVID‐19 confirmed case on March 10, 2020.

**Table 1:** Laboratory results for the mother and neonate

Date	Days after birth	Laboratory test	Value	Reference range^a)^
Mother				
February 8, 2020	–	NAT of throat swab	−	−
February 26, 2020	–	Leucocyte count [× 10^9^ L^−1^]	8.11	3.5–9.5
		Lymphocyte count [× 10^9^ L^−1^]	1.56	1.1–3.2
		Neutrophil ratio [%]	75.7	40–75
		Hb [g L^−1^]	124	>110
		PLT [× 10^9^ L^−1^]	181	150–400
		Fibrinogen [mg L^−1^]	4.24	2–4
		D‐dimer [µg mL^−1^]	1.7	<0.4
		CRP [mg L^−1^]	0.7	<1
February 27, 2020	–	Leucocyte count [× 10^9^ L^−1^]	11.37	3.5–9.5
		Lymphocyte count [× 10^9^ L^−1^]	1.46	1.1–3.2
		Neutrophil ratio [%]	83	40–75
		Hb [g L^−1^]	112	>110
		PLT [× 10^9^ L^−1^]	147	150–400
		Fibrinogen [mg L^−1^]	5.99	2–4
		D‐dimer [µg mL^−1^]	4.14	<0.4
		CRP [mg L^−1^]	13	<1
Neonate				
February 27, 2020	0	NAT of amniotic fluid	−	−
		NAT of throat swab	−	−
		SARS‐CoV‐2 IgG [AU mL^−1^]	134.13	≤10
		SARS‐CoV‐2 IgM [AU mL^−1^]	3.52	≤10
		Urine leucocyte	+	−
		Specific gravity	1.009	1.001–1.020
		Leucocyte count [µL^−1^]	20.7	0–4 per low‐power field
		Leucocyte count [× 10^9^ L^−1^]	16.48	9–30
		Neutrophil count [× 10^9^ L^−1^]	11.15	2.5–8
		Neutrophil ratio [%]	67.7	55–70
		Lymphocyte ratio [%]	20.8	20–40
		RBC [× 10^12^ L^−1^]	3.74	4.8–7.1
		Hb [g L^−1^]	140	140–240
		PLT [× 10^9^ L^−1^]	369	150–300
		β_2_ microglobulin [mg L^−1^]	1.55	1–2
		Urine creatinine [µmol L^−1^]	2677	88–176 µmol kg^−1^ d^−1^
		Cystatin C [mg L^−1^]	1.87	0.6–2.5
		hs‐CRP [mg L^−1^]	0.5	<10
		cTnI [pg mL^−1^]	8.1	<20
		NT‐proBNP [pg mL^−1^]	1342	<125 (adult)
		TBil [µmol L^−1^]	76.4	17.1–205
		DBil [µmol L^−1^]	7.5	1.7–5.1
		ALT [U L^−1^]	6	4–36
		AST [U L^−1^]	26	35–140
		TP [g L^−1^]	51.4	46–74
		ALB [g L^−1^]	35.3	35–54
March 4, 2020	6	SARS‐CoV‐2 IgG [AU mL^−1^]	215.04	≤10
		SARS‐CoV‐2 IgM [AU mL^−1^]	2.38	≤10
		NAT of throat swab	−	−
		Stool routine	−	−
		Urinalysis	−	−
March 8, 2020	10	SARS‐CoV‐2 IgG [AU mL^−1^]	119	≤10
		SARS‐CoV‐2 IgM [AU mL^−1^]	2.79	≤10
		NAT of throat swab	−	−
		NAT of anal swab	−	−
		NAT of urine	−	−
		NAT of excrement	−	−
		TBil [µmol L^−1^]	128.4	17.1–205
		DBil [µmol L^−1^]	14	1.7–5.1
		ALT [U L^−1^]	6	4–36
		AST [U L^−1^]	18	15–60
		TP [g L^−1^]	49	46–74
		ALB [g L^−1^]	32.8	35–54
		LDH [U L^−1^]	295	160–450
		hsCRP [mg L^−1^]	0.3	<10
		Leucocyte count [× 10^9^ L^−1^]	9.87	9–30
		Neutrophil count [× 10^9^ L^−1^]	4.44	2.5–8
		Neutrophil ratio [%]	45	55–70
		Lymphocyte count [× 10^9^ L^−1^]	3.94	1–4
		Lymphocyte ratio [%]	39.9	20–40
		RBC [× 10^12^ L^−1^]	3.15	4.8–7.1
		Hb [g L^−1^]	112	140–240
		PLT [× 10^9^ L^−1^]	546	150–300
		IL‐1β [pg mL^−1^]	<5	<5
		IL‐2R [U mL^−1^]	1374	223–710
		IL‐6 [pg mL^−1^]	<1.5	<8.5
		IL‐8 [pg mL^−1^]	12.7	<62
		IL‐10 [pg mL^−1^]	<5	<7
		TNF‐α [pg mL^−1^]	14.4	<3.5

Notes: NAT, nucleic acid test; SARS‐CoV‐2, severe acute respiratory syndrome coronavirus 2; RBC, red blood cell; Hb, hemoglobin; PLT, platelet; CRP, C reaction protein; hsCRP, hypersensitivity C reaction protein; cTnI, cardiac troponin I; NT‐proBNP, N‐terminal pronatriuretic peptide; TBiL, total bilirubin; DBiL, direct bilirubin; ALT, alanine aminotransferase; AST, aspartate aminotransferase; TP, total protein; ALB, albumin; IL, interleukin; TNF, tumor necrosis factor; −, negative; +, positive.

^a)^ Pagana K. Mosby's Manual of Diagnostic and Laboratory Tests, Mosby, 2017.

The throat swabs, peripheral blood samples, and amniotic fluid of the neonate were collected and tested for nucleic acid and antibodies of SARS‐CoV‐2 immediately at birth. The only positive result was high IgG level in peripheral blood (134.13 AU mL^−1^). Most of the laboratory findings were normal except an elevated leukocyte count and an alleviated lymphocyte (listed in Table 1). The antibodies in neonate peripheral blood were tested twice on March 4 and March 8, 2020, and the IgG was still at a high level (215.04 and 119 AU mL^−1^). However, nucleic acid tests on the throat swab, anal swab, excrement, and urine at the same time were all negative. On March 8, 2020, the neonate developed symptoms of jaundice and the laboratory findings showed an elevated serum total bilirubin, interleukin 2 receptor, and tumor necrosis factor α (listed in Table 1). The neonate was diagnosed as physiological jaundice not neonatal hyperbilirubinemia by his medical history and the clinical standard.^[^ ^5^ ^]^ In this case, the neonate jaundice appeared on the 10th day after birth, and lasted for 4 d. The direct bilirubin was 14 µmol L^−1^, which was less than the minimum threshold of neonatal hyperbilirubinemia, i.e., 34 µmol L^−1^. An intermittent phototherapy was provided. The neonate recovered and was discharged on March 11, 2020. A seven‐month follow‐up showed that the development of neonate, including the intelligence, weight (11 kg), and height (80 cm), were all in the normal range. Reexaminations of the mother's SARS‐CoV‐2 NATs and antibodies also came negative on May 7, 2020.

COVID‐19 is an emerging disease which is highly contagious and spreading rapidly all over the world. Pregnant women and infants are potential vulnerable groups owing to the physiological changes and low immune, respectively. Several studies^[^ ^3^, ^6^, ^7^ ^]^ revealed that pregnant women may have a high risk of obstetric complications and perinatal adverse outcomes after virus infection.

In this case, we reported a pregnant woman exposed to SARS‐CoV‐2 in her late trimester. She was finally diagnosed as a COVID‐19 confirmed case by serum antibodies on March 10, 2020. According to the previous studies which demonstrated that the antibodies levels increased rapidly during the first two weeks,^[^ ^8^, ^9^ ^]^ we retrospect her course of the disease and speculate that she was infected before delivery. Her nucleotide assays were all negative, considering the possibility of sample collection and/or detection methods and the positive antibodies. Given that she developed fever symptom on February 2, 2020, and the abnormity in her CT on February 8, there is a high possibility that she was infected at that time. Therefore, the infant may be exposed to SARS‐CoV‐2 for 26 d.

The mother and the newborn recovered well till the censorship of follow‐up. Disease was mild in this patient and the symptom improved rapidly only by taking Ibuprofen suspension, which demonstrated that pregnant women with COVID‐19 might not have adverse outcomes. Our multicenter retrospective study reviewed 92 pregnant women with COVID‐19 and their 78 newborns, and all the pregnant women had recovered and discharged from hospital (unpublished data). Large series are required to confirm the outcome of this cohort and the effect of the virus on the fetus.

This patient was exposed to virus at 34+1 weeks’ gestation and gave birth at 37+5 weeks. Positive IgG and negative IgM were found in three times’ tests of antibodies for SARS‐CoV‐2 in the neonate. Due to the nucleic acid of infant's throat swabs, excrement, anal swab and amniotic fluid were all negative, the possibility of perinatal transmission of COVID‐19 was low. Therefore, it is possible that mother produced antibodies against the virus, and the IgG was transferred to the fetus via placenta, which may protect the fetus/neonate. There is no evidence to demonstrate that COVID‐19 causes neonatal jaundice. In our case, the neonate was diagnosed with physiological jaundice according to his medical history and laboratory findings. In fact, neonatal jaundice is one of the most common phenomena in neonatal period, and mainly associated with the metabolic characteristics of bilirubin.^[^ ^10^ ^]^ The newborn had no pathological jaundice without cause of hyperbilirubinemia, for example, perinatal asphyxia, sepsis, hepatitis, biliary atresia, choledochal cyst, metabolic acidosis, immune hemolysis, glucose‐6‐phosphate dehydrogenase (G6PD) deficiency, and drugs.^[^ ^5^ ^]^ However, during the COVID‐19 outbreak period, some rapid and progressive newborn diseases like bilirubin encephalopathy should not be ignored. Obstetricians and pediatricians should review the maternal medical history and birth history in detail, and examine comprehensively.

Clinicians should always weight the balance between possible risk of vertical transmission and the benefit of expectant management in pregnancy. As there was no direct evidence of COVID‐19 vertical transmission, the patient with stable vital signs and normal fetal ultrasound chose expectant management for 26 d. The neonate's birth weight was in a normal range and no complications and abnormal symptoms were noticed except a high IgG titer. Currently, no definitive conclusion has been reached on when to induce birth with mild COVID‐19. In this case, the development of the neonate was satisfactory and the mother recovered well, which indicated that immediate termination of pregnancy might not be necessary for all cases. Continued expectation to deliver at term instead of preterm can decrease the potential risk of severe perinatal and infant complications and is beneficial to the development of the neonate.^[^ ^11^, ^12^ ^]^

## Conflict of Interest

The authors declare no conflict of interest.

## Author Contributions

R.L. and D.W. contributed equally to this work. D.R.D. and S.J.L. contributed to study design. R.L., D.W., X.G.L., D.L., R.J.W., and L.J. contributed to the data collection. R.L. and D.W. analyzed the data. R.L. and W.L.L. interpreted the results. R.L., D.R.D., W.L.L., and S.J.L. wrote the manuscript. All authors contributed to the revision of the manuscript and the final approval of the version to be published.
